# The Improvement Effects of Virtual Reality-Assisted Exercise Training on Motor and Psychiatric Symptoms in Patients With Parkinson’s Disease: A Meta-Analysis of Randomized Controlled Trials

**DOI:** 10.62641/aep.v54i4.2301

**Published:** 2026-08-15

**Authors:** Hanwen Zhang, Xiaolei Yuan

**Affiliations:** ^1^Department of Neurology, Longhua Hospital Affiliated to Shanghai University of Traditional Chinese Medicine, 200032 Shanghai, China

**Keywords:** Parkinson’s disease, virtual reality, motor function, psychiatric symptoms, meta-analysis

## Abstract

**Background:**

This study aimed to systematically evaluate the combined improvement effects of virtual reality (VR)-assisted exercise training on motor function and psychiatric symptoms in patients with Parkinson’s disease (PD), and to provide more scientific evidence-based support for precise clinical rehabilitation.

**Methods:**

This study searched China National Knowledge Infrastructure (CNKI), Wanfang, China Science and Technology Journal Database (VIP Database), PubMed, Web of Science and Scopus for randomized controlled trials (RCTs) examining VR interventions in patients with idiopathic PD covering the period from database inception to 1 March 2026. Two investigators independently performed literature screening and data extraction. Methodological quality was assessed using the Cochrane Risk of Bias Tool 2.0. Meta-analysis was conducted using Review Manager 5.4 and R 4.6.0 software.

**Results:**

A total of 37 RCTs involving 1749 patients with PD were included. Meta-analysis results showed that, compared with conventional rehabilitation training, VR-assisted training significantly improved motor outcomes, as reflected by the Berg Balance Scale (BBS; mean difference (MD) = 3.96), Timed Up and Go Test (TUGT; MD = –2.48), and Unified Parkinson’s disease Rating Scale Part III (UPDRS-III; MD = –2.94). Regarding psychiatric symptoms, VR training significantly enhanced specific cognitive function (Montreal Cognitive Assessment (MoCA); MD = 1.94), alleviated subjective depressive mood (Beck Depression Inventory (BDI); MD = –1.75), and improved activity-specific balance confidence (Activities-specific Balance Confidence Scale (ABC); MD = 12.03). However, no statistically significant between-group differences were found in global cognition (Mini-Mental State Examination (MMSE)) and observer-rated depression (Hamilton Depression Rating Scale (HAMD)).

**Conclusions:**

VR-assisted exercise training effectively improves comprehensive motor function, specific cognition and depressive mood in patients with PD. Therapeutic efficacy may be influenced by the immersion level of equipment and intervention duration. VR confers short-term improvements, but its long-term effects remain unknown. It may serve as a promising adjunctive therapy; however, routine clinical recommendation requires further evidence from extended follow-up studies. High-quality RCTs with prolonged follow-up periods are still warranted to clarify the long-term effects of VR interventions with varying immersion levels.

## Introduction

Parkinson’s disease (PD) is a prevalent, chronic, progressive neurodegenerative disorder characterized predominantly by motor symptoms such as bradykinesia, dystonia, resting tremor and postural instability, and frequently accompanied by non-motor manifestations such as cognitive decline and depression [1, 2].As the disease advances, patients experience progressive deterioration in balance and activities of daily living, which elevates fall risk and substantially diminishes quality of life [3].

Currently, pharmacological treatment and deep brain stimulation are the primary clinical interventions; however, their efficacy in improving postural control, balance function and certain non-motor symptoms remains limited. Furthermore, long-term application is associated with attenuated therapeutic response and adverse effects [4, 5]. Although conventional rehabilitation training has been demonstrated to positively affect motor function in PD, it imposes substantial demands on therapist expertise, patient financial resources and training facilities. Consequently, patients frequently struggle with long-term adherence, resulting in generally low compliance [6].

In recent years, virtual reality (VR) technology has emerged as a novel rehabilitation tool and has been progressively integrated into neurorehabilitation. VR can deliver immersive or non-immersive multisensory interactive environments, support high-intensity, task-oriented training and enhance patient engagement and treatment adherence through real-time feedback [7, 8].

Existing studies have indicated that VR training can improve balance function and activities of daily living in stroke survivors, cognitive function in the elderly, and motor symptoms in patients with PD [9, 10]. However, the current evidence remains inconsistent. One systematic review found that VR only positively affects gait speed and step length in PD, without remarked improvement in balance [11], another study has reported that VR combined with conventional rehabilitation is superior to conventional treatment alone in improving motor function and quality of life [12].

Furthermore, existing randomized controlled trials (RCTs) exhibit considerable heterogeneity in sample size, intervention protocols, outcome measures and VR equipment types, making it difficult to reach unified conclusions.

Although several meta-analyses have examined the effects of VR on motor function in patients with PD, most have focused on single outcome measures (e.g., balance or gait) and lack a systematic, integrated analysis of motor function and psychiatric symptoms (e.g., cognitive function, depressive state) [13, 14, 15]. In particular, high-quality evidence on the combined effects of VR training on activities of daily living, cognitive and emotional state in patients with PD remains scarce. Moreover, the influence of VR types, intervention dosages and control interventions on therapeutic efficacy remains unclear.

Therefore, this systematic review and meta-analysis aimed to comprehensively evaluate the effects of VR-assisted exercise training on motor function and psychiatric symptoms in patients with PD, and to explore potential influencing factors, thereby providing more robust scientific evidence for clinical rehabilitation practice.

## Materials and Methods

### Search Strategy

This study systematically searched China National Knowledge Infrastructure (CNKI), Wanfang, China Science and Technology Journal Database (VIP Database), PubMed, Web of Science, and Scopus for articles published from database inception to 1 March 2026. The search strategy utilized Boolean operators “OR” and “AND” to combine two sets of keywords. Chinese search terms (in Pinyin) were as follows: Set 1 keywords included “xuni xianshi (VR)”, “xuni xianshi fuzhu yundong xunlian (VR-assisted exercise training)” and “youxi (game)”; set 2 keywords included “pajinsen bing” and “pajinsen huanzhe”. English search terms were as follows: Set 1 keywords included “Virtual reality”, “Virtual reality-assisted exercise training”, “VR” and “video game”; set 2 keywords included “Parkinson”, “Parkinson disease” and “parkinson’s disease”. Additionally, this study manually screened the reference lists of relevant systematic reviews and retrieved studies to avoid omissions.

A complete PubMed search strategy is provided below as an example; search strings for other databases were adapted accordingly.

PubMed search strategy: (Parkinson disease OR Parkinson OR Parkinson’s disease) AND (Virtual Reality OR virtual reality OR VR OR video game OR exergaming) AND (randomized controlled trial OR controlled clinical trial OR randomized OR placebo OR randomly OR trial).

### Study Selection

The inclusion criteria were as follows: (1) Participants: Patients with a confirmed clinical diagnosis of idiopathic PD based on the UK Parkinson’s Disease Society Brain Bank criteria (for international studies) or the Chinese Guidelines for the Diagnosis and Treatment of Parkinson’s Disease (for Chinese studies) [16, 17]. No restrictions were imposed on age, sex, disease duration, or severity, given that current evidence suggests VR training may be benefit patients across different stages, and available RCTs have not systematically excluded patients based on disease duration. (2) Intervention: The experimental group received VR-based interventions, including immersive or non-immersive formats. For the purpose of this review, VR was defined as a computer-based simulation enabling real-time interaction with a virtual environment through multisensory feedback. (3) Comparator: The control group received conventional treatment, traditional physical therapy, or other non-VR interventions (e.g., pharmacotherapy, neuromodulation). (4) Outcomes: Outcome measures included assessments of depressive symptoms (Hamilton Depression Rating Scale (HAMD) and Beck Depression Inventory (BDI)), cognitive function (Mini-Mental State Examination (MMSE) and Montreal Cognitive Assessment (MoCA)), balance confidence (Activities-specific Balance Confidence Scale (ABC)), and motor function (Berg Balance Scale (BBS), Timed Up and Go Test (TUGT), and Unified Parkinson’s Disease Rating Scale Part III (UPDRS-III)). (5) Study Design: RCTs. A study was identified as an RCT if authors explicitly stated random assignment using terms such as “randomized”, “random allocation” or “randomly assigned.” Quasi-randomized trials (e.g., allocation by date of birth or medical record number) were excluded. (6) Studies were required to report sufficient data for effect size calculation (e.g., means and standard deviations (SDs), standard errors, 95% confidence intervals (CIs), median, interquartile range (IQR)) [18]. Published journal articles and master’s/doctoral theses were eligible. Language was restricted to Chinese and English. The exclusion criteria were as follows: (1) Reviews, conference abstracts, case reports, animal experiments and basic research. (2) Non-randomized controlled trials. (3) Studies with incomplete data, inaccessible full texts, or duplicate publications.

### Data Extraction

Two researchers independently performed data extraction using a pre-designed standardized form, with disagreements resolved through discussion or consultation with a third researcher. Extracted content primarily included: first author, publication year, country or region, study design type, sample size, detailed intervention protocols for the experimental and control groups (including VR type, intervention dosage, frequency and duration), as well as pre- and post-intervention means and SD for all outcome measures (e.g., HAMD, MMSE, BBS), along with group sample sizes.

### Quality Assessment

Two researchers independently assessed methodological quality using the Cochrane risk-of-bias tool for randomized trials (RoB 2.0, Cochrane, London, UK), as recommended in the Cochrane Handbook for Systematic Reviews of Interventions (version 6.5) [19]. The RoB 2.0 assesses five specific domains: (1) bias arising from the randomization process; (2) bias due to deviations from intended interventions; (3) bias due to missing outcome data; (4) bias in outcome measurement; and (5) bias in selection of the reported result. Each domain was rated as “low risk”, “some concerns” or “high risk” according to the standardized RoB 2.0 algorithms. After completing the assessments independently, the two researchers resolved discrepancies through discussion to reach consensus. In cases of persistent disagreement, a third researcher was consulted. Overall risk of bias was summarized based on domain-level judgments.

### Data Analysis

Statistical analyses were performed using Review Manager 5.4 (Cochrane, Copenhagen, Denmark) and R 4.6.0 (R Core Team/R Foundation, Vienna, Austria) software. As all outcome measures were continuous variables, effect size was expressed as the mean difference (MD) with 95% CI. For studies reporting continuous outcomes as median and IQR, sample mean and SD were estimated using the method proposed by Wan *et al*. [18], as recommended by the Cochrane Handbook for converting medians and IQRs to means and SDs for meta-analysis purposes. Specifically, the sample mean was estimated as the median (assuming approximate symmetry), and the SD was estimated as SD = IQR/1.35, using R software (version 4.6.0). Heterogeneity was assessed using the Q-test and the I^2^ statistic. If I^2^ ≤ 50% and *p* ≥ 0.1, indicating low heterogeneity, a fixed-effects model was used for the meta-analysis. If I^2^ > 50% or *p* < 0.1, indicating substantial heterogeneity, a random-effects model was applied. Sensitivity analyses were conducted by sequentially excluding individual studies and re-running the meta-analysis to evaluate the robustness of pooled results. Publication bias was assessed using funnel plots, with symmetric funnel plots considered indicative of the absence of significant publication bias. In addition, for outcomes with ≥10 studies, Egger’s linear regression test was performed to quantify small-study effects (*p* < 0.05 considered significant). For outcomes with <10 studies, Egger’s test was not conducted due to low statistical power. The significance level for all statistical tests was set at α = 0.05 (two-tailed).

## Results

### Basic Characteristics of Included Studies

**Fig. 1.**
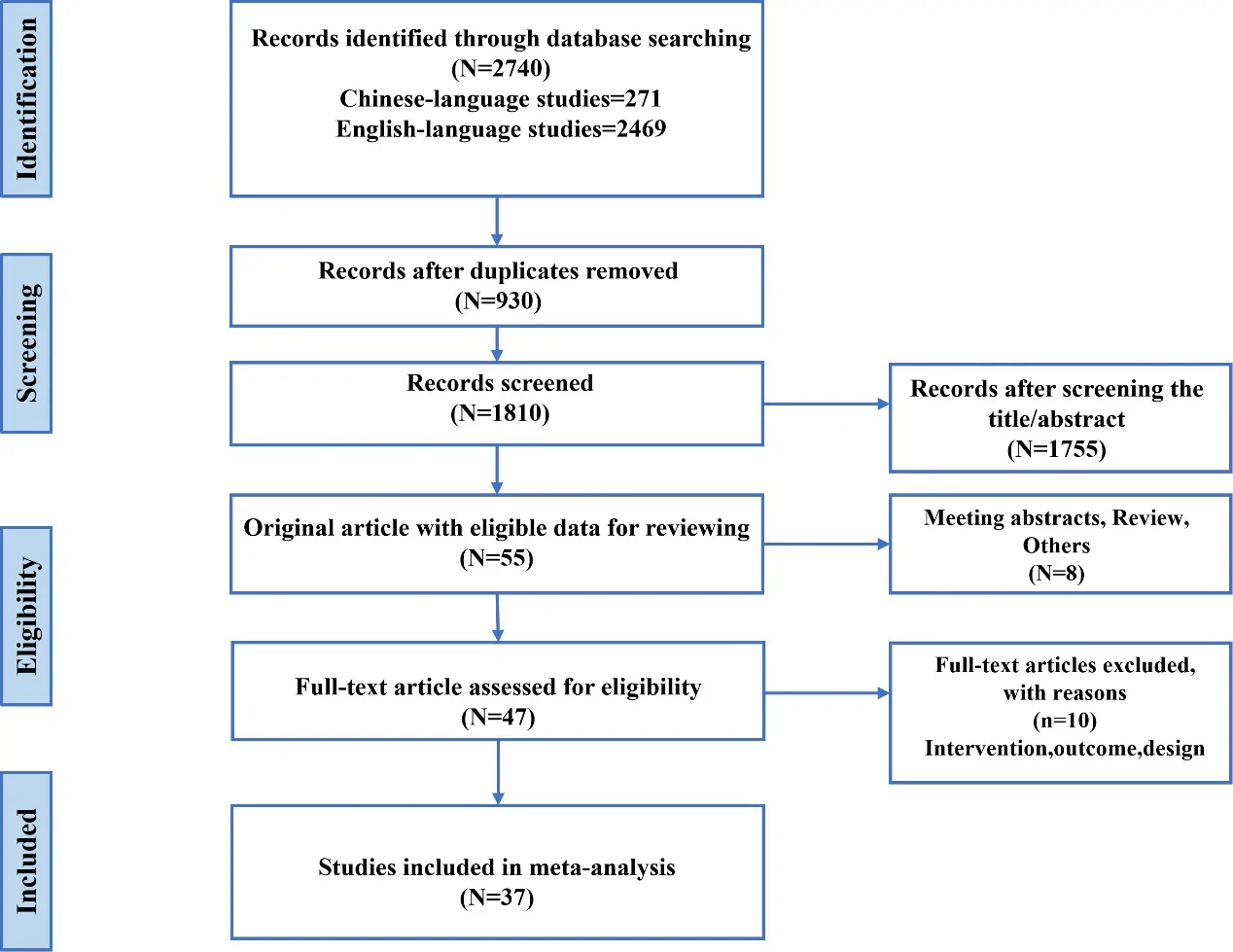
**Flow diagram of literature screening**.

**Fig. 2.**
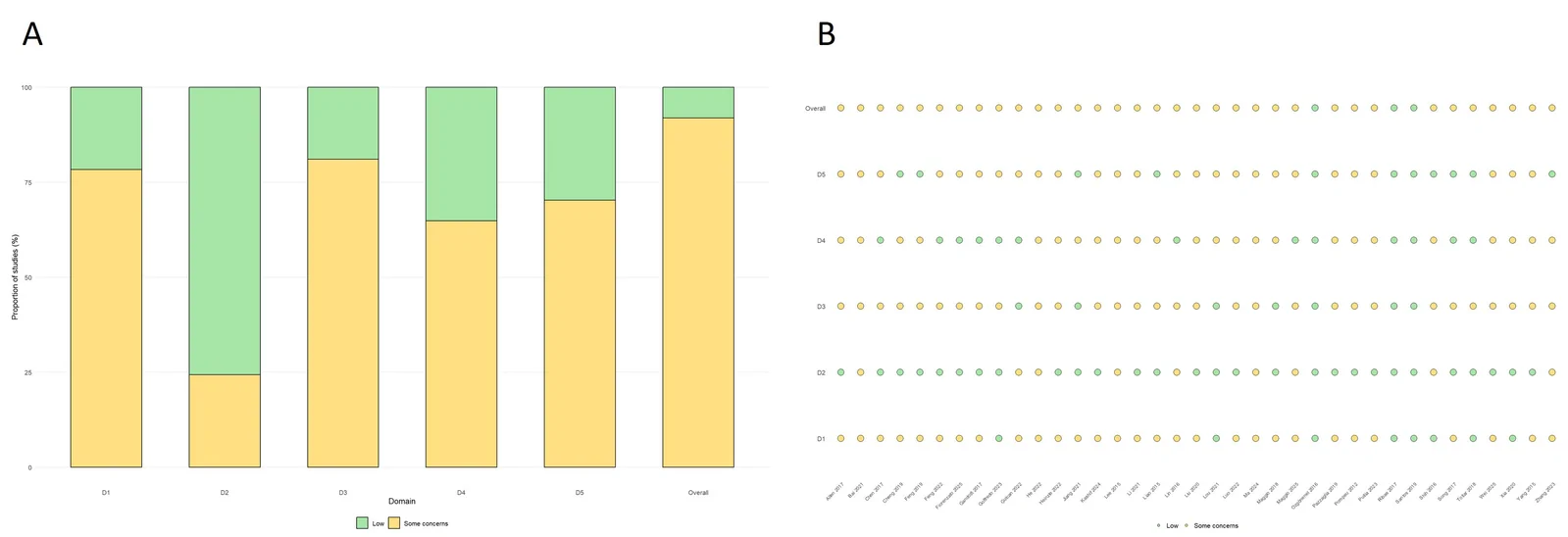
**Risk of bias assessment using the RoB 2.0 tool**. (A) Weighted bar plot illustrating the percentage of studies classified as low risk (green) or some concerns (yellow) for each domain (D1–D5) and overall. (B) Traffic-light plot with circles showing domain-level judgments for each individual study. Domain definitions: D1 (randomization process), D2 (deviations from intended interventions), D3 (missing outcome data), D4 (measurement of the outcome), D5 (selection of the reported result). Overall risk was determined as “some concerns” if any domain had some concerns, otherwise “low risk”. No study was judged as high risk.

A total of 2740 records were retrieved from the databases within the search period. After removing duplicates and screening titles and abstracts, followed by full-text review, 37 studies were ultimately included. The detailed literature screening process is presented in Fig. 1. Among the included studies, 19 were conducted in China, with the remainder distributed across Turkey (n = 2), Italy (n = 7), Pakistan (n = 1), Austria (n = 1), Brazil (n = 3), Australia (n = 2), Korea (n = 1), and Hungary (n = 1). A total of 1749 participants were included in the analysis. All studies employed VR technology for the intervention groups, while control groups received conventional training or standard rehabilitation. Intervention duration ranged from 4 to 12 weeks. Regarding outcome measures, 24 studies reported BBS, 15 reported TUGT, 13 reported UPDRS-III, 9 reported MoCA, 4 reported BDI, and 3 studies each reported HAMD, MMSE and ABC. Baseline characteristics are summarized in Table 1 (Ref. [20, 21, 22, 23, 24, 25, 26, 27, 28, 29, 30, 31, 32, 33, 34, 35, 36, 37, 38, 39, 40, 41, 42, 43, 44, 45, 46, 47, 48, 49, 50, 51, 52, 53, 54, 55, 56]). Risk of bias assessment using RoB 2.0 tool is presented in Fig. 2. Most studies had some concerns in the randomization process (D1) due to insufficient reporting, and a small proportion had some concerns in the domain of bias due to deviations from intended interventions (D2) owing to the nature of VR interventions. No study was rated as high risk in any domain, and all studies were classified as having some concerns overall. The detailed risk of bias assessment is presented graphically in Fig. 2.

**Table 1.** **Characteristics of included studies**.

Study	Country	Sample size (T/C)	Intervention group measures	Control group measures	Duration	Outcome events	Outcome variables
Wei *et al*. [20]	China	55/55	conventional rehabilitation + loaded core muscle training + VR	conventional rehabilitation + loaded core muscle training	8 weeks	motor function	UPDRS-III
Zhang *et al*. [21]	China	32/32	conventional rehabilitation + rTMS + VR	conventional rehabilitation + rTMS + conventional cognitive training	8 weeks	cognitive function; motor function	MMSE, MoCA, UPDRS-III
Ma [22]	China	40/40	conventional rehabilitation + rTMS + VR	conventional rehabilitation + rTMS + conventional cognitive training	4 weeks	cognitive function; motor function	MMSE, MoCA, UPDRS-III
Li [23]	China	24/24	Wii VR	conventional rehabilitation	8 weeks	balance; mobility; cognitive function; depression	BBS, MoCA (2005 version), HAMD, TUGT
Lou *et al*. [24]	China	57/56	VR-based caren balance training	conventional balance training	3 months	balance	BBS
Feng *et al*. [25]	China	34/34	pramipexole hydrochloride tablets + VR	pramipexole hydrochloride tablets	12 weeks	depression	HAMD, BDI
Luo *et al*. [26]	China	16/16	conventional rehabilitation + VR	conventional rehabilitation + conventional cognitive training	4 weeks	cognitive function	MoCA
Liu *et al*. [27]	China	21/21	VR	conventional balance training	4 weeks	balance	BBS
Lin *et al*. [28]	China	17/14	VR	conventional balance training	4 weeks	balance; mobility	BBS, TUGT
Cheng *et al*. [29]	China	20/20	conventional rehabilitation + VR	conventional rehabilitation	8 weeks	balance; mobility	BBS, TUGT
Xia *et al*. [30]	China	15/15	conventional rehabilitation + VR	conventional rehabilitation	4 weeks	motor function; balance; mobility; cognitive function	UPDRS-III, BBS, TUGT, MoCA
Chen *et al*. [31]	China	23/23	VR	conventional rehabilitation	6 weeks	motor function; balance; mobility; depression	UPDRS-III, BBS, TUGT, HAMD
Jiang [32]	China	38/38	trunk core muscle strengthening + VR	trunk core muscle strengthening	1 month	balance	BBS
He and Jiang [33]	China	41/41	progressive rehabilitation nursing + VR	progressive rehabilitation nursing	1 month	balance	BBS
Bai [34]	China	42/42	strengthening exercise + VR	strengthening exercise	1 month	motor function; balance	UPDRS-III, BBS
Feng *et al*. [35]	China	14/14	conventional rehabilitation + VR	conventional rehabilitation	12 weeks	mobility; motor function; balance	TUGT, UPDRS-III, BBS
Gulcan *et al*. [36]	Turkey	15/15	conventional rehabilitation + VR	conventional rehabilitation	6 weeks	motor function; balance; mobility; balance confidence	UPDRS-III, BBS, TUGT, ABC
Goffredo *et al*. [37]	Italy	49/48	conventional rehabilitation + VR	conventional rehabilitation	30 sessions	motor function; mobility	UPDRS-III (MDS-UPDRS-III), TUGT
Kashif *et al*. [38]	Pakistan	20/20	conventional rehabilitation + VR	conventional rehabilitation	12 weeks	motor function; balance; balance confidence	UPDRS-III, BBS, ABC
Gandolfi *et al*. [39]	Italy	36/34	conventional rehabilitation + VR	conventional rehabilitation	7 weeks	balance; balance confidence	BBS, ABC
Pullia *et al*. [40]	Italy	10/10	conventional rehabilitation + VR	conventional rehabilitation	5 weeks	balance; mobility; motor function	BBS, TUGT, UPDRS-III (MDS-UPDRS-III)
Pazzaglia *et al*. [41]	Italy	25/26	conventional rehabilitation + VR	conventional rehabilitation	6 weeks	balance	BBS
Yang *et al*. [42]	China	11/12	conventional rehabilitation + VR	conventional rehabilitation	6 weeks	balance; mobility; motor function	BBS, TUGT, UPDRS-III
Fiorenzato *et al*. [43]	Italy	15/15	VR	conventional rehabilitation	4 weeks	depression	BDI
Maggio *et al*. [44]	Italy	10/10	VR	traditional cognitive training	8 weeks	cognitive function	MoCA (2005 version)
Liao *et al*. [45]	China	12/12	Wii VR	conventional rehabilitation	6 weeks	mobility	TUGT
Heinzle *et al*. [46]	Austria	29/29	VR	conventional rehabilitation	4 weeks	motor function	UPDRS-III
Pompeu *et al*. [47]	Brazil	16/16	VR	conventional rehabilitation	7 weeks	balance; cognitive function	BBS, MoCA
Maggio *et al*. [48]	Italy	10/10	VR	traditional cognitive training	8 weeks	cognitive function	MMSE
Song *et al*. [49]	Australia	28/25	VR	conventional rehabilitation	12 weeks	mobility; cognitive function	TUGT, MoCA (version 7.1)
Allen *et al*. [50]	Australia	18/19	VR	conventional rehabilitation	12 weeks	cognitive function	MoCA
Lee *et al*. [51]	Korea	10/10	VR	conventional rehabilitation	6 weeks	balance; depression	BBS, BDI
Santos *et al*. [52]	Brazil	13/14	VR	conventional rehabilitation	8 weeks	balance; mobility	BBS, TUGT
Ribas *et al*. [53]	Brazil	10/10	VR	conventional rehabilitation	12 weeks	balance	BBS
Shih *et al*. [54]	China	10/10	VR	conventional rehabilitation	8 weeks	balance; mobility	BBS, TUGT
Özgönenel *et al*. [55]	Turkey	15/18	conventional rehabilitation + VR	conventional rehabilitation	5 weeks	balance; mobility	BBS, TUGT
Tollár *et al*. [56]	Hungary	25/25	VR	conventional rehabilitation	5 weeks	balance; depression	BBS, BDI

Note: T, intervention group; C, control group; VR, virtual reality; rTMS, repetitive transcranial magnetic stimulation; Wii VR, Nintendo Wii-based exergaming (a non-immersive virtual reality system using motion-sensing controllers and balance board); UPDRS-Ⅲ, Unified Parkinson’s Disease Rating Scale Part III; MMSE, Mini-Mental State Examination; MoCA, Montreal Cognitive Assessment; BBS, Berg Balance Scale; TUGT, Timed Up and Go Test; HAMD, Hamilton Depression Rating Scale; BDI, Beck Depression Inventory; ABC, Activities-specific Balance Confidence Scale. Scale versions are reported in the “Outcome Variables” column only when explicitly stated in the original study. The absence of a version number indicates that the original study did not specify the version.

### Meta-Analysis Results

#### BBS

**Fig. 3.**
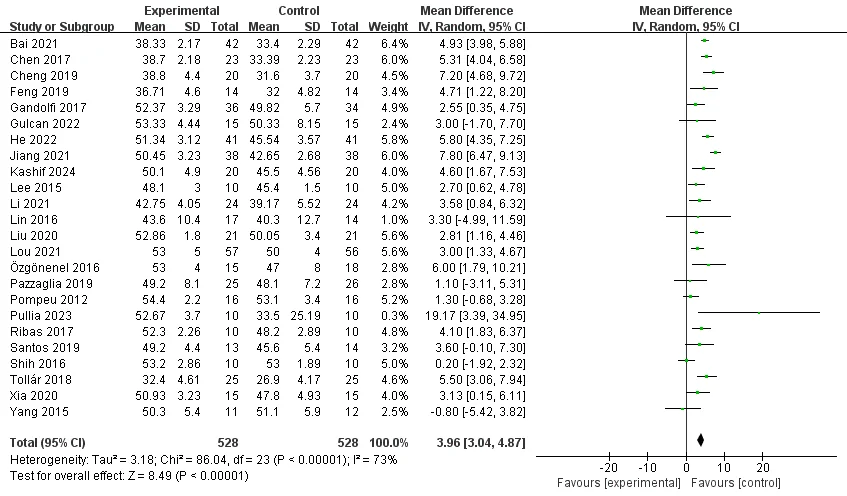
**Forest plot of the effect of virtual reality-assisted exercise training versus conventional rehabilitation on BBS scores in patients with Parkinson’s disease**. SD, standard deviation; CI, confidence interval; BBS, Berg Balance Scale.

**Fig. 4.**
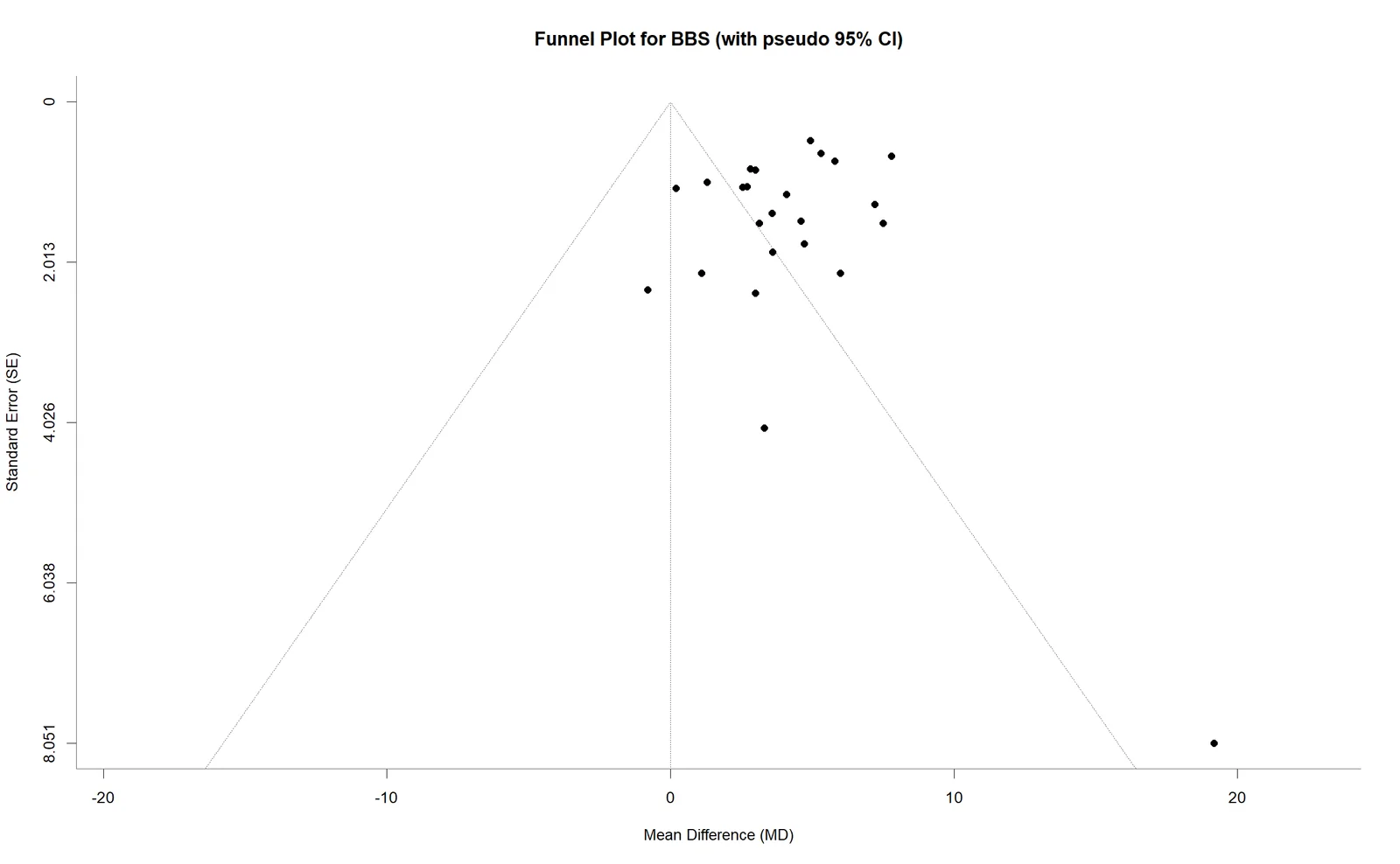
**Funnel plot for the assessment of publication bias in the meta-analysis of virtual reality-assisted exercise training on BBS scores in patients with Parkinson’s disease**. SE, standard error; MD, mean difference; BBS, Berg Balance Scale.

A total of 24 studies were included, comprising 528 participants in the experimental group and 528 in the control group. Given that I^2^ > 50%, a random-effects model was employed. Pooled results indicated that VR-assisted exercise training significantly improved BBS scores in patients with PD (MD = 3.96, 95% CI: 3.04 to 4.87, Z = 8.49, *p* < 0.00001), indicating superiority over conventional rehabilitation training in improving balance (Fig. 3). The funnel plot suggested the possible publication bias (Fig. 4).

#### TUGT

**Fig. 5.**
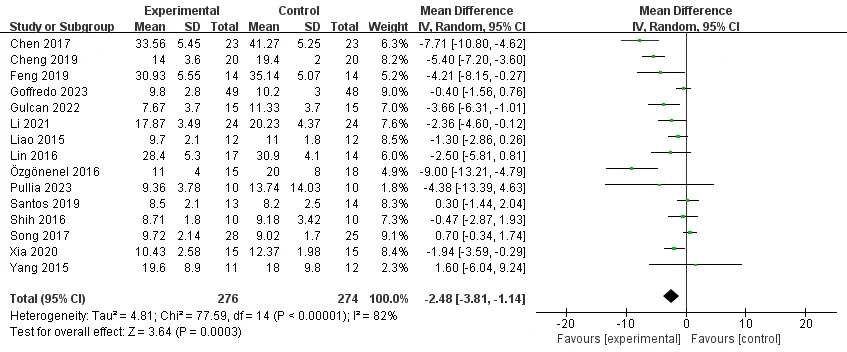
**Forest plot of the effect of virtual reality-assisted exercise training versus conventional rehabilitation on TUGT in patients with Parkinson’s disease**. SD, standard deviation; CI, confidence interval; TUGT, Timed Up and Go Test.

**Fig. 6.**
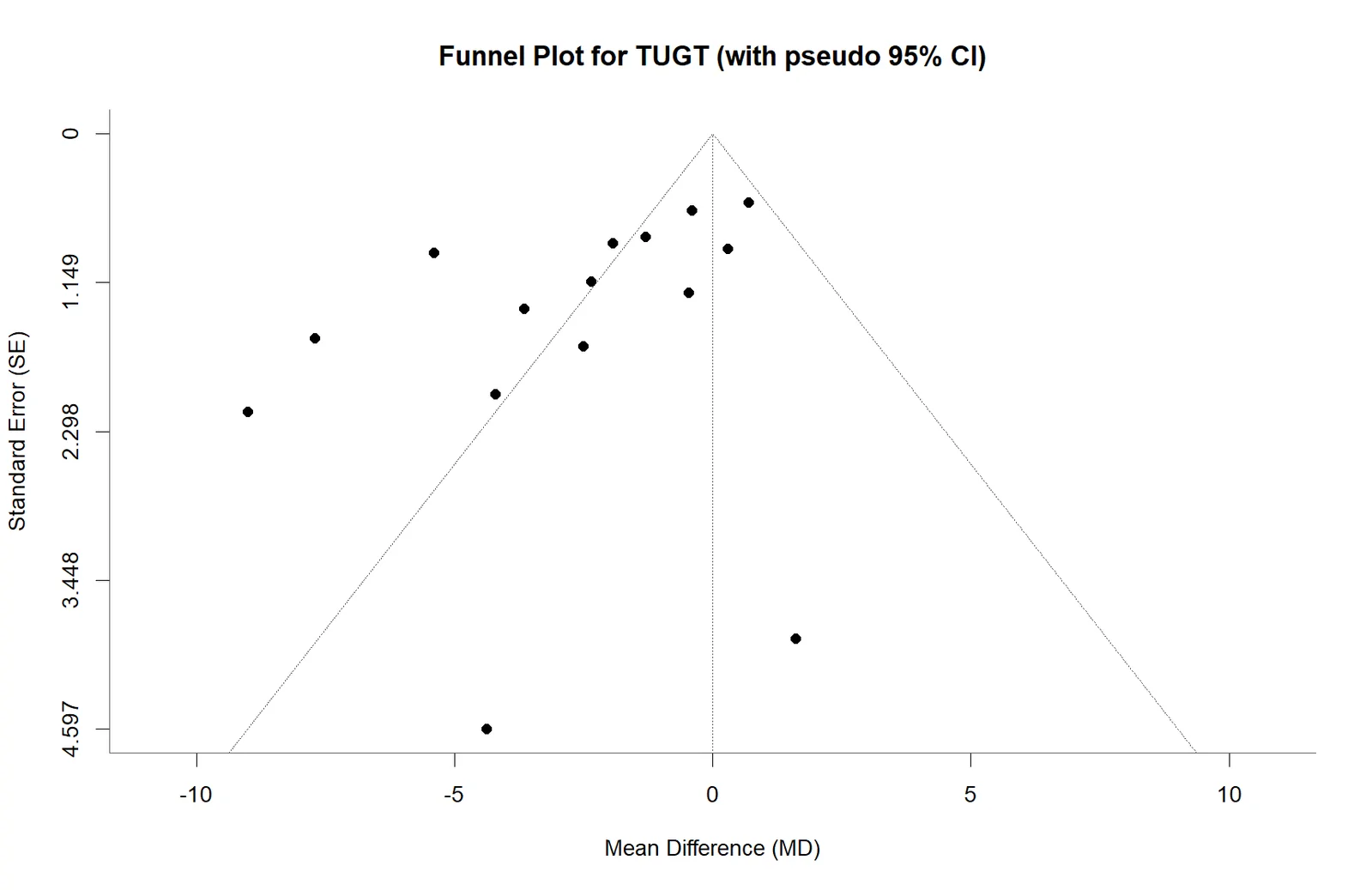
**Funnel plot for the assessment of publication bias in the meta-analysis of virtual reality-assisted exercise training on TUGT in patients with Parkinson’s disease**. SE, standard error; MD, mean difference; TUGT, Timed Up and Go Test.

A total of 15 studies involving 550 patients with PD were included. Due to the presence of high heterogeneity (I^2^ = 82%, Chi^2^ = 77.59, df = 14, *p* < 0.00001, Tau^2^ = 4.81), a random-effects model was employed. Pooled results indicated that VR-assisted exercise training significantly shortened TUGT completion time (MD = -2.48, 95% CI: -3.81 to -1.14, Z = 3.64, *p *= 0.0003), demonstrating superiority over conventional rehabilitation training in improving mobility (Fig. 5). The funnel plot suggested possible publication bias (Fig. 6).

#### UPDRS-III

**Fig. 7.**
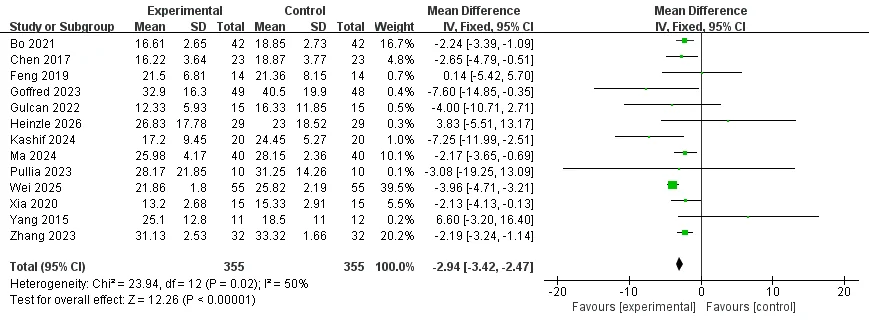
**Forest plot of the effect of virtual reality-assisted exercise training versus conventional rehabilitation on UPDRS-Ⅲ in patients with Parkinson’s disease**. SD, standard deviation; CI, confidence interval; UPDRS-Ⅲ, Unified Parkinson’s Disease Rating Scale Part III.

**Fig. 8.**
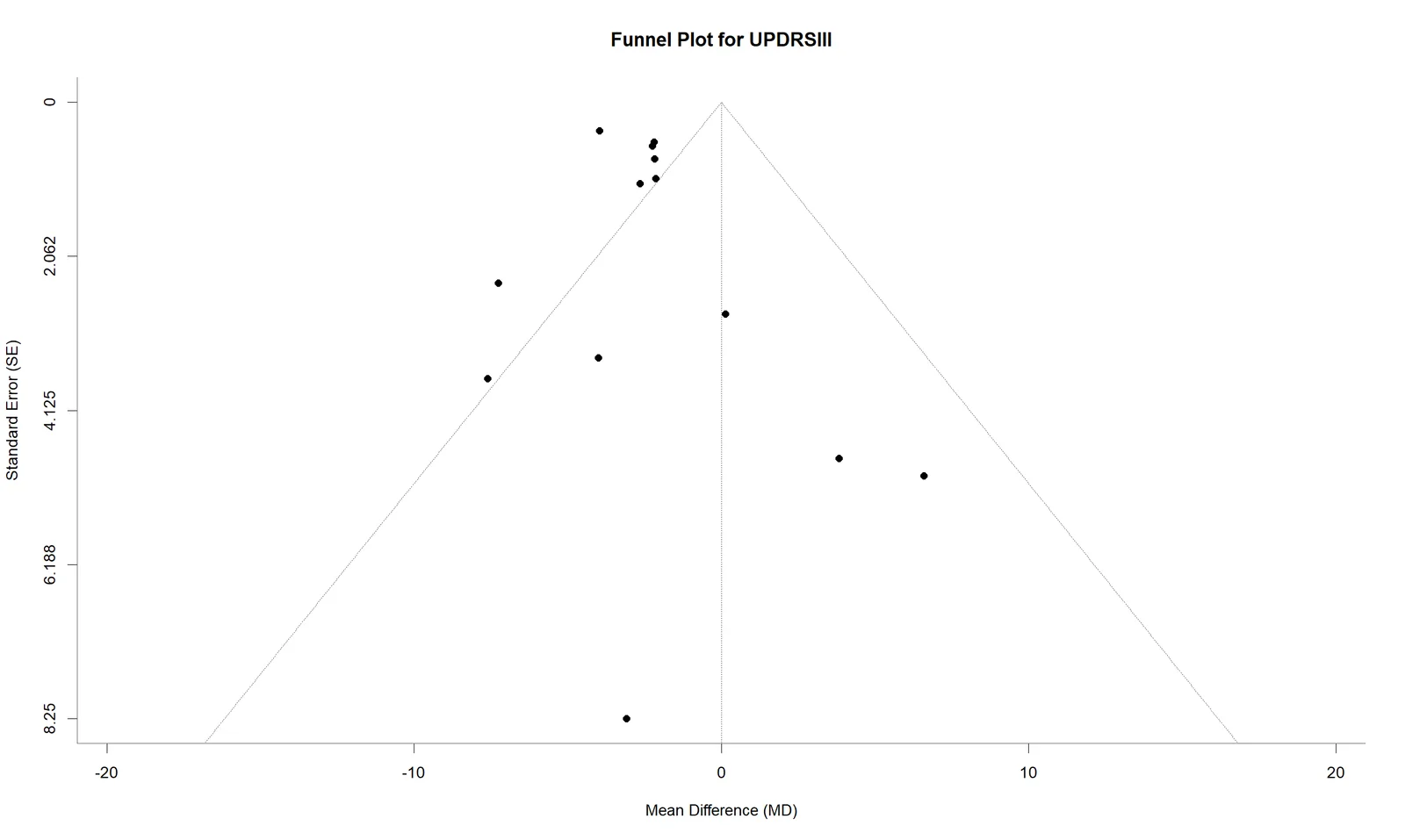
**Funnel plot for the assessment of publication bias in the meta-analysis of virtual reality-assisted exercise training on UPDRS-Ⅲ in patients with Parkinson’s disease**. SE, standard error; MD, mean difference; UPDRS-Ⅲ, Unified Parkinson’s Disease Rating Scale Part III.

A total of 13 studies involving 710 patients with PD were included. Heterogeneity testing revealed I^2^ = 50%, Chi^2^ = 23.94, df = 12, *p *= 0.02, and a fixed-effects model was therefore employed. Pooled results indicated that VR-assisted exercise training significantly reduced UPDRS-III scores (MD = -2.94, 95% CI: -3.42 to -2.47, Z = 12.26, *p* < 0.00001), demonstrating superiority over conventional rehabilitation training in improving motor function (Fig. 7). The funnel plot was largely symmetric, suggesting a low risk of publication bias (Fig. 8).

#### HAMD

**Fig. 9.**
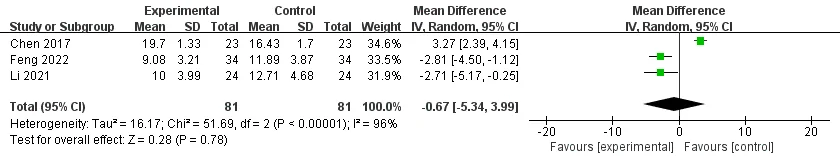
**Forest plot of the effect of virtual reality-assisted exercise training versus conventional rehabilitation on HAMD in patients with Parkinson’s disease**. SD, standard deviation; CI, confidence interval; HAMD, Hamilton Depression Rating Scale.

A total of 3 studies were included, comprising 81 participants in the experimental group and 81 in the control group. Due to the presence of extremely high heterogeneity (I^2^ = 96%, Chi^2^ = 51.69, df = 2, *p* < 0.00001, Tau^2^ = 16.17), a random-effects model was employed. Pooled results revealed no significant difference between VR-assisted exercise training and conventional rehabilitation training in improving HAMD scores (MD = -0.67, 95% CI: -5.34 to 3.99, *p *= 0.78, Fig. 9). Given the small number of included studies, a funnel plot was not constructed.

#### MMSE

**Fig. 10.**
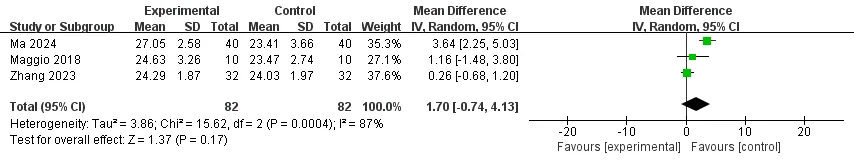
**Forest plot of the effect of virtual reality-assisted exercise training versus conventional rehabilitation on MMSE in patients with Parkinson’s disease**. SD, standard deviation; CI, confidence interval; MMSE, Mini-Mental State Examination.

A total of 3 studies were included, comprising 82 participants in the experimental group and 82 in the control group. Due to the presence of high heterogeneity (I^2^ = 87%, Chi^2^ = 15.62, df = 2, *p *= 0.0004, Tau^2^ = 3.86), a random-effects model was employed. Pooled results revealed no significant difference between VR-assisted exercise training and conventional rehabilitation training in improving MMSE scores (MD = 1.70, 95% CI: -0.74 to 4.13, *p *= 0.17, Fig. 10). Given the small number of included studies, a funnel plot was not constructed.

#### MoCA

**Fig. 11.**
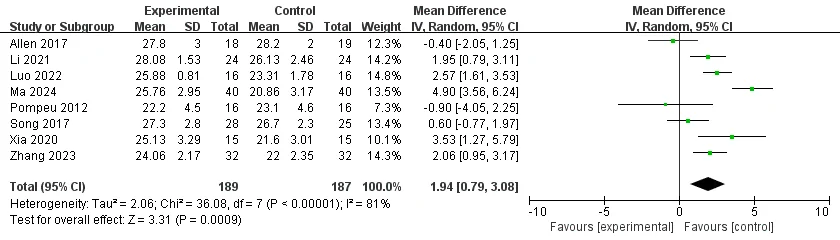
**Forest plot of the effect of virtual reality-assisted exercise training versus conventional rehabilitation on MoCA in patients with Parkinson’s disease**. SD, standard deviation; CI, confidence interval.

A total of 9 studies were initially included; however, because the control group standard deviation was zero and thus inestimable in Maggio *et al*. [44], data from 8 studies were ultimately incorporated, comprising an estimated 189 participants in the experimental group and 187 in the control group. Given the presence of high heterogeneity (I^2^ = 81%, Chi^2^ = 36.08, df = 7, *p* < 0.00001, Tau^2^ = 2.06), a random-effects model was employed. Pooled results indicated that VR-assisted exercise training significantly improved MoCA scores in patients with PD (MD = 1.94, 95% CI: 0.79 to 3.08, Z = 3.31, *p *= 0.0009), demonstrating superiority over conventional rehabilitation training in enhancing cognitive function (Fig. 11). Given the small number of included studies, a funnel plot was not constructed.

#### BDI

**Fig. 12.**
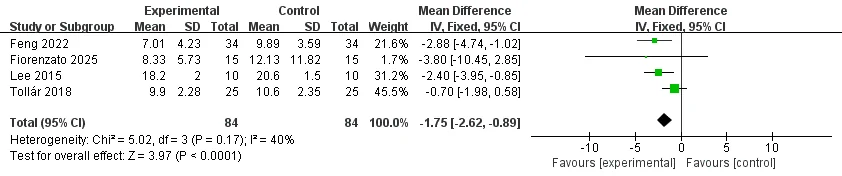
**Forest plot of the effect of virtual reality-assisted exercise training versus conventional rehabilitation on BDI in patients with Parkinson’s disease**. SD, standard deviation; CI, confidence interval; BDI, Beck Depression Inventory.

A total of 4 studies were included, comprising 84 participants in the experimental group and 84 in the control group. Heterogeneity testing revealed I^2^ = 40%, Chi^2^ = 5.02, df = 3, *p *= 0.17, and a fixed-effects model was therefore employed. Pooled results indicated that VR-assisted exercise training significantly reduced BDI scores in patients with PD (MD = -1.75, 95% CI: -2.62 to -0.89, *p* < 0.0001), demonstrating superiority over conventional rehabilitation training in alleviating depressive symptoms (Fig. 12). Given the small number of included studies, a funnel plot was not constructed.

#### ABC

**Fig. 13.**
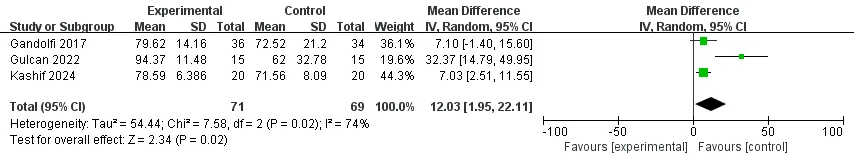
**Forest plot of the effect of virtual reality-assisted exercise training versus conventional rehabilitation on ABC in patients with Parkinson’s disease**. SD, standard deviation; CI, confidence interval; ABC, Activities-specific Balance Confidence Scale.

A total of 3 studies were included, comprising 71 participants in the experimental group and 69 in the control group. Given the presence of substantial heterogeneity (I^2^ = 74%), a random-effects model was employed. Pooled results indicated that VR-assisted exercise training significantly improved ABC scores in patients with PD (MD = 12.03, 95% CI: 1.95 to 22.11, *p *= 0.02), demonstrating superiority over conventional rehabilitation training in enhancing balance confidence (Fig. 13). Given the small number of included studies, a funnel plot was not constructed.

### Sensitivity Analysis and Publication Bias

Leave-one-out sensitivity analyses were performed for all outcome measures to assess whether any single study disproportionately influenced the pooled effect estimates. Results indicated that no single study dominated the overall effect sizes for most outcomes, demonstrating robustness of the findings. The leave-one-out forest plots for all outcomes are provided in the Supplementary Materials (**Supplementary Figs. 1–8**). For the HAMD outcome, the pooled result changed from non-significant to significant after excluding the study by Chen *et al*. [31] (MD = -2.78, 95% CI: -4.17 to -1.38, *p* < 0.0001). This instability is likely attributable to the small number of included studies (n = 3) and the relatively short intervention duration and higher baseline HAMD scores in Chen *et al*. [31]. Therefore, the HAMD finding should be interpreted with caution. For the MMSE outcome, the pooled result also changed from non-significant to significant after excluding the study by Zhang *et al*. [21] (MD = 2.66, 95% CI: 0.29 to 5.04, *p *= 0.03). This occurred because Zhang *et al*. [21] showed a minimal between-group difference (MD = 0.26) with relatively small SD, contributing a high weight but a near-zero effect, thereby diluting the overall pooled estimate. Therefore, the MMSE finding should also be interpreted with caution.

Egger’s regression test was performed for outcomes with ≥10 studies (i.e., BBS, TUGT, UPDRS-III, Table 2). For BBS (24 studies), Egger’s test revealed significant small-study effects (intercept = 2.3638, *t* = 2.1392, *p* = 0.0438), which is consistent with the asymmetry observed in the funnel plot (Fig. 4), indicating potential publication bias. For UPDRS-III (13 studies), the test was also non-significant (intercept =-1.02, *t* = -1.08, *p *= 0.30). In contrast, for TUGT (15 studies), Egger’s test showed a significant intercept (intercept = -6.13, *t* = -4.34, *p* < 0.001), indicating the presence of small-study effects or potential publication bias. Therefore, the pooled TUGT effect should be interpreted with caution. For the remaining outcomes (e.g., HAMD, MMSE, MoCA, BDI, ABC), the number of included studies was <10; thus, Egger’s test was not performed due to insufficient statistical power. Publication bias for these outcomes could not be reliably assessed, and the results should be considered exploratory.

**Table 2.** **Egger’s regression test for publication bias**.

Outcome	Number of studies	Intercept	SE of intercept	*t*-value	*p*-value
BBS	24	2.3638	1.105	2.1392	0.0438
TUGT	15	−6.13	1.41	−4.34	<0.001
UPDRS-III	13	−1.02	0.94	−1.08	0.3

SE, standard error; BBS, Berg Balance Scale; TUGT, Timed Up and Go Test; UPDRS-III, Unified Parkinson’s Disease Rating Scale Part III.

## Discussion

This systematic review, which conducted a comprehensive meta-analysis of published RCTs incorporating 37 studies with 1749 patients with PD, evaluated the effect of VR-assisted exercise training on motor function and psychiatric symptoms. The results demonstrated that, compared with conventional rehabilitation training, VR training significantly improved motor function as measured by BBS (24 studies, 1056 participants; MD = 3.96, 95% CI: 3.04 to 4.87), TUGT (15 studies, 550 participants; MD = -2.48, 95% CI: -3.81 to -1.14) and UPDRS-III (13 studies, 710 participants; MD = -2.94, 95% CI: -3.42 to -2.47). Regarding psychiatric symptom-related outcomes, VR training significantly improved MoCA scores (8 studies, 376 participants; MD = 1.94, 95% CI: 0.79 to 3.08), BDI (4 studies, 168 participants; MD = -1.75, 95% CI: -2.62 to -0.89) and ABC (3 studies, 140 participants; MD = 12.03, 95% CI: 1.95 to 22.11). However, no significant superiority over conventional rehabilitation training was observed for MMSE or HAMD. Overall, VR-assisted exercise training demonstrates positive effects on motor function, cognitive performance, depressive symptoms, and activity-specific balance confidence in patients with PD.

### Improvement Effects of VR Training on Motor Function and Underlying Mechanisms

This study found that VR training significantly improved BBS scores (MD = 3.96, 95% CI: 3.04 to 4.87). This finding partially aligns with previous systematic reviews. For instance, Dockx *et al*. [11] concluded that VR can improve balance in patients with PD. The magnitude of BBS improvement observed in this study (3.96 points) falls below the minimal detectable change, which is typically considered to be 5 points [57]. Therefore, whether this improvement reaches a clinically meaningful threshold remains uncertain. Furthermore, Egger’s test indicated significant small-study effects for BBS (*p* = 0.0438), suggesting that the pooled estimate may be upwardly biased due to the preferential publication of studies with positive findings; consequently, the true effect size could be smaller than observed.

Functional magnetic resonance imaging (FMRI) evidence suggests that VR-based motor rehabilitation induces cortical reorganization, primarily involving the primary motor cortex, sensorimotor cortex, and supplementary motor area, which is associated with functional improvement in patients with neurological disorders [58]. Additionally, VR-based task-oriented repetitive training enhances synaptic plasticity and accelerates neural compensation [59], while trial-by-trial feedback and motivating stimuli within the virtual environment may enhance motivation and facilitate learning [47].

This study also demonstrated that VR significantly improved TUGT completion time (MD = -2.48 seconds) and UPDRS-III scores (MD = -2.94 points) in patients with PD, indicating that VR training effectively enhances comprehensive mobility functions such as rising from a chair, walking, and turning, as well as overall motor function. These results are consistent with the meta-analyses conducted by Wang *et al*. [60] and Triegaardt *et al*. [61]. Notably, heterogeneity for UPDRS-III was relatively low (I^2^ = 50%), suggesting stable findings across studies. Whether the TUGT improvement (2.48 seconds) is clinically meaningful should be interpreted in the context of the minimal clinically important difference (MCID) for patients with PD (commonly considered 1.5 to 2.5 seconds). The observed improvement in this study approximates or reaches this threshold.

It is noteworthy that a recent systematic review and meta-analysis by Ishaq *et al*. [62] specifically investigated head-mounted (immersive) VR rehabilitation in PD and reported significant benefits in quality of life (9-item Parkinson’s Disease Questionnaire (PDQ-39)) but did not find statistically significant improvements in UPDRS-III or the TUG single-task. The discrepancy between their findings and our robust results (which show significant improvements in UPDRS-III and TUGT) can be explained by several methodological differences. Firstly, the current meta-analysis included a broader scope of VR modalities, predominantly non-immersive and semi-immersive systems (e.g., Kinect, Wii, C-Mill), which heavily involve gross motor movements, dynamic balance, and spatial navigation, directly translating to better TUGT and UPDRS-III performance. In contrast, fully immersive head-mounted devices obscure the real-world view, which may restrict patients from performing large-scale dynamic movements due to safety or fall-risk concerns, potentially limiting transfer to clinically assessed motor outcomes. Secondly, this analysis strictly pooled data from 37 RCTs (1749 participants), whereas the meta-analysis for TUG and UPDRS-III in Ishaq *et al*. [62] relied on pre-post data from a much smaller sample (e.g., only 54 participants across 3 studies for UPDRS-III), limiting statistical power and increasing the risk of type II errors. Notably, the pooled effect for UPDRS-III in their study (MD = -2.64, 95% CI: -5.72 to 0.45) was directionally consistent with our finding (MD = -2.94, 95% CI: -3.42 to -2.47) but did not reach significance, likely due to insufficient sample size. Both reviews agree that VR training improves quality of life and balance-related outcomes (e.g., Tinetti Scale) in PD. These findings extend the evidence by demonstrating that VR-assisted exercise training—particularly when task-specific balance and gait exercises are incorporated—is effective for motor function. Future head-to-head trials directly comparing immersive versus non-immersive VR for motor outcomes in PD are warranted.

### Improvement Effects of VR Training on Psychiatric Symptoms

VR training significantly improved MoCA scores (MD = 1.94), whereas no significant effect was observed for MMSE. This discrepancy may be attributable to the differing sensitivities of the two instruments: MoCA is more sensitive to PD-related mild cognitive impairment, particularly in domains such as executive function and attention, whereas MMSE is weighted toward global cognition and memory and may be less responsive to cognitive changes in patients with PD [63]. Furthermore, only three studies reported MMSE data, and the small sample size may have contributed to a false-negative finding. The mechanisms by which VR enhances cognition may include: reduced compensatory frontal lobe activation and improved cognitive efficiency through multisensory stimulation [64]; and the sense of immersion experienced during VR training, which enhances attention and memory, thereby positively influencing overall cognitive function [65]. Additionally, VR training significantly improved ABC scores (MD = 12.03, 95% CI: 1.95 to 22.11), indicating enhanced balance confidence, which may have practical implications for reducing fear of falling and increasing willingness to engage in activities.

VR training significantly reduced BDI scores (MD = -1.75, 95% CI: -2.62 to -0.89, *p* < 0.0001), suggesting a beneficial effect on self-reported depressive symptoms. However, no significant difference was found for HAMD (MD = -0.67, 95% CI: -5.34 to 3.99, *p *= 0.78), which is a clinician-rated scale. Although both instruments measure depression, BDI places greater emphasis on self-reported affective and somatic symptoms, whereas HAMD requires skilled interview techniques. Moreover, heterogeneity for HAMD was extremely high (I^2^ = 96%), and the sensitivity analysis showed that the result was not robust (excluding one study changed the significance). Given the small number of included studies (n = 3) and the high heterogeneity, current evidence does not support a definitive conclusion that VR training improves clinician-rated depression. Future well-powered RCTs are needed to clarify this issue.

### Heterogeneity Analysis

In the present meta-analysis, substantial heterogeneity was observed for several outcome measures (e.g., TUGT I^2^ = 82%; MoCA I^2^ = 81%). Potential sources of heterogeneity may include: (1) differences in VR equipment type (immersive vs. non-immersive; commercial gaming consoles vs. specialized rehabilitation platforms); (2) variations in intervention dosage (session duration: 20–60 minutes, frequency: 2–5 sessions per week, total intervention duration: 4–12 weeks); (3) inconsistency in control interventions (conventional rehabilitation, conventional cognitive training, sham intervention); and (4) differences in disease severity among patients. Previous studies have also noted that commercial gaming systems (e.g., Nintendo Wii), owing to their non-personalized design, may yield inconsistent effects [66], whereas specialized VR platforms tend to exhibit lower heterogeneity. Despite recognizing these potential sources, this study was unable to conduct formal subgroup analyses or meta-regression to quantitatively explore their effect. The primary reasons are: (i) the included studies reported VR device types, intervention dosages, control conditions, and disease severity inconsistently, often lacking sufficient detail to allow reliable categorization; (ii) many subgroups contained too few studies (e.g., only 3 studies for ABC, 3 for MMSE) to yield meaningful statistical power; and (iii) heterogeneity in outcome measures (e.g., different versions of the same scale) further precluded pooling within narrow subgroups. Therefore, the unexplained heterogeneity remains a major limitation of this meta-analysis, and these findings should be interpreted as preliminary evidence requiring confirmation in future standardized trials.

### Limitations

Several limitations of this study should be acknowledged. Firstly, the included trials had methodological limitations mainly due to the lack of blinding of participants and therapists, which is inherently difficult to achieve in VR interventions. According to the RoB 2.0 tool, this resulted in some concerns in the bias due to deviations from intended interventions domain (D2) for most studies; however, all studies were at low risk of bias in the other four domains. No study was judged as high risk overall. Secondly, substantial heterogeneity was observed for several outcome measures, and as noted above, this study could not clarify its sources through subgroup analyses or meta-regression, which limits the generalizability of the findings. Thirdly, none of the included studies incorporated long-term follow-up data (≥6 months), as the intervention periods ranged only from 4 to 12 weeks; consequently, the sustainability of VR training effects remains undetermined. Additionally, although the inclusion criteria did not restrict disease duration, all identified RCTs had intervention periods of only 2–12 weeks, reflecting the current state of evidence in this emerging field; thus, the sustainability of VR effects across different disease stages remains unknown. Fourthly, funnel plot asymmetry was observed for BBS and TUGT, and Egger’s regression test confirmed significant small-study effects for both BBS (*p* = 0.0438) and TUGT (*p* < 0.001), indicating a risk of publication bias for both outcomes. Therefore, the pooled BBS and TUGT effect estimates may be inflated and should be interpreted with caution. Fifthly, the cost-effectiveness of VR interventions was not assessed, as only a few studies reported cost information, leaving the economic feasibility of VR technology in this context unclear. Finally, due to the diversity of outcome measures and the limited number of studies available for certain outcomes (e.g., ABC, HAMD), the results for these specific indicators should be interpreted with caution.

### Future Research Direction

Future research should prioritize large sample, multicentre, rigorously designed RCTs, with particular efforts directed toward implementing blinding of therapists and outcome assessors to mitigate potential bias. Furthermore, it is essential to explore the optimal intervention dosage of VR training, including the ideal combination of frequency, intensity, and duration, and to compare the differential therapeutic effects of immersive versus non-immersive VR systems. Given the current absence of long-term observational data, subsequent trials should incorporate extended follow-up periods of no less than six months to ascertain the durability of VR-induced improvements and to evaluate their effect on hard endpoints such as fall rates and hospitalization incidence. Additionally, the inclusion of cost-effectiveness analyses within the study framework is recommended to provide health economic evidence for clinical implementation and healthcare policy decisions. Finally, conducting stratified analyses based on specific Hoehn and Yahr stages will facilitate the development of individualized and precise rehabilitation strategies for patients with PD.

## Conclusions

This meta-analysis demonstrates that VR-assisted exercise training, as an innovative rehabilitation modality, offers certain advantages in improving comprehensive symptoms in patients with PD. Compared with conventional rehabilitation training, VR training effectively improves motor function (as measured by BBS, TUGT, and UPDRS-III) as well as some psychiatric symptoms (as reflected in MoCA, ABC and BDI scores). However, current evidence does not yet indicate that VR training is significantly superior to conventional interventions in terms of MMSE and HAMD outcomes.

Based on a synthesis of the available literature, the clinical efficacy of VR technology may be influenced by factors such as the VR equipment type and intervention duration. Some external studies have suggested a potential advantage of high-immersion devices in short-term interventions, but direct comparative evidence from this meta-analysis is lacking, as no subgroup analysis by immersion level was feasible.

In clinical practice, VR-assisted training may be considered as a supplementary approach to traditional rehabilitation therapy for PD, with potential short-term benefits in improving patient engagement and adherence. However, due to the absence of long-term follow-up data (≥6 months) and the substantial heterogeneity across existing studies, a strong clinical recommendation cannot be made at this stage. Future high-quality RCTs with large samples, rigorous blinding, and extended follow-up (≥6 months) are needed to determine the long-term effects, optimal dosage, and cost-effectiveness of VR training in PD.

## Availability of Data and Materials

The datasets used and analysed during the current study were available from the corresponding author on reasonable request.

## Supplementary Material

Supplementary material associated with this article can be found, in the online version, at https://doi.org/10.62641/aep.v54i4.2301.
